# Cell viability and electrical response of breast cancer cell treated in aqueous graphene oxide solution deposition on interdigitated electrode

**DOI:** 10.1038/s41598-021-00171-3

**Published:** 2021-10-19

**Authors:** Muhammad M. Ramli, A. S. Rosman, N. S. Mazlan, M. F. Ahmad, D. S. C. Halin, R. Mohamed, Nurul H. Osman, Ali H. Reshak

**Affiliations:** 1 Faculty of Electronic Engineering Technology, Universiti Malaysia Perlis (UniMAP), Pauh Putra Campus, 02600 Kangar, Perlis Malaysia; 2 Geopolymer & Green Technology, Centre of Excellence (CEGeoGTech), Universiti Malaysia Perlis (UniMAP), Pauh Putra Campus, 02600 Kangar, Perlis Malaysia; 3 Regenerative Medicine Cluster, Advance Medical and Dental Institute, Universiti Sains Malaysia, Bertam, 13200 Kepala Batas, Pulau Pinang Malaysia; 4 Applied Electromagnetic Laboratory 1, Department of Physics, Faculty of Science, Universiti Putra Malaysia, 43400 Serdang, Selangor Malaysia; 5 Physics Department, College of Science, University of Basrah, 61004 Basrah, Iraq; 6 Department of Instrumentation and Control Engineering, Faculty of Mechanical Engineering, Czech Technical Universiti in Prague, Technicka 4, Prague 6, 166 07 Czech Republic

**Keywords:** Biotechnology, Cancer, Materials science

## Abstract

Breast cancer is one of the most reported cancers that can lead to death. Despite the advances in diagnosis and treatment procedures, the possibility of cancer recurrences is still high in many cases. With that in consideration, researchers from all over the world are showing interest in the unique features of Graphene oxide (GO), such as its excellent and versatile physicochemical properties, to explore further its potential and benefits towards breast cancer cell treatment. In this study, the cell viability and electrical response of GO, in terms of resistivity and impedance towards the breast cancer cells (MCF7) and normal breast cells (MCF10a), were investigated by varying the pH and concentration of GO. Firstly, the numbers of MCF7 and MCF10a were measured after being treated with GO for 24 and 48 h. Next, the electrical responses of these cells were evaluated by using interdigitated gold electrodes (IDEs) that are connected to an LCR meter. Based on the results obtained, as the pH of GO increased from pH 5 to pH 7, the number of viable MCF7 cells decreased while the number of viable MCF10a slightly increased after the incubation period of 48 h. Similarly, the MCF7 also experienced higher cytotoxicity effects when treated with GO concentrations of more than 25 µg/mL. The findings from the electrical characterization of the cells observed that the number of viable cells has corresponded to the impedance of the cells. The electrical impedance of MCF7 decreased as the number of highly insulating viable cell membranes decreased. But in contrast, the electrical impedance of MCF10a increased as the number of highly insulating viable cell membranes increased. Hence, it can be deduced that the GO with higher pH and concentration influence the MCF7 cancer cell line and MCF10a normal breast cell.

## Introduction

The research on Graphene Oxide (GO) involved in medical applications has increased constantly throughout the years. The medical applications of GO include drug delivery^1,2^, tissue engineering^3^, diagnostics and cancer treatment^4,5^. The interactions between graphene-based nanomaterials with biological systems were previously discovered and explored^1,5,6^. However, there is still a lack of scientific support to determine the cytotoxicity effects caused by nanomaterials towards the normal and cancer cells. Hence, the crucial mechanisms of nanotoxicity due to the production of reactive oxygen species (ROS) were studied^3,7^. When the ROS is over generated, it also creates oxidative stress that leads to the failure of the cells to maintain the normal physiological redox-regulated functions. This consequently resulted in DNA damage, imbalance of cell signals and changes in cells cytotoxicity, apoptosis and cancer initiation. By comparing the normal and cancer cells, the ROS production and oxidative stresses are much higher in the cancer cells, making them more likely to undergo cell mutations, uncontrolled initiation of cellular proliferation, genetic instability and cell death. Therefore, the potential of graphene-based biomaterials to inhibit cancer that can limit ROS production, as these have the potential to affect the cell viability of cancerous cells^3^. According to Ref.^8^, even though graphene or graphene-based nanomaterials exhibited no effect on the mammalian cells at low concentrations, but high graphene concentrations can affect the cell membrane integrity and cause cell death. These findings were similar and were also supported by Refs.^4,5^. These studies suggested that the interactions between the high concentrations of graphene or graphene-based materials with the cells can degrade the cell membrane integrity, including the early-stage breast cancer cells (MCF7), pancreatic cancer cells (Panc-1), and invasive breast cancer cells (MDA-MB-231).

Most commonly used biosensors are affinity-based. The affinity-based biosensor is referred to as an immobilized capture probe that binds to a particular target, therefore causing physiochemical changes that can be detected by a transducer^9^. So that the cancer diagnosis can be made in a short time. The interdigitated electrode (IDEs) is one of the promising alternatives to assimilate the biosensors into a portable lab-on-chip device due to its ability to detect electrical changes in terms of impedance, resistance or capacitance^10^. As stated by Ref.^11^, IDEs are usually used for various applications, especially as biological and chemical sensors, due to the low cost of production, ease of fabrication and high sensitivity.

The research by Ref.^12^ illustrated the principles of electrochemical impedance spectroscopy (EIS) measurement during the presence and absence of cells on the Bode impedance spectra. When there are no cells attached, the impedance will only be obtained from the resistance of the solution, R_sol_ and the double-layer capacitance of the electrode, C_dl_. However, when cells are deposited on the electrode surface, the newly formed equivalent circuit will include the impedance inclusive of the resistance of the dropped cells, R_cell_ and its capacitance, C_cell_ attached in parallel. The presence of cells also influences the changes of interface impedance, where the impedance will increase due to the insulating cells membranes. This eventually triggers the ionic environment changes around the electrode or solution interfaces, thus leading to the changes in R_sol_ and C_dl_ values. As stated in Eqs. (1) and (2), the capacitance (C_cell_) that contributes to the impedance (Z″) is depending on the frequency (f), while the resistance (R_cell_) that contributes to the impedance (Z′) is independent of the frequency. Hence, when the cells are attached at the electrode surface, the new magnitude impedance, Z_cell_ that consists of R_cell_ and C_cell_ is generated, as shown in Eq. (3).

1 $${Z}^{^{\prime\prime} }= \frac{1}{2\pi f{C}_{cell}},$$

2 $${Z}^{^{\prime}}={R}_{cell},$$

3 $${Z}_{cell}= \sqrt{{{R}^{2}}_{cell }+ \frac{1}{{(2\pi f{C}_{cell})}^{2}}},$$

4 $$\varnothing = {\mathrm{tan}}^{-1}\left(\frac{-1}{2\pi f{R}_{cell}{C}_{cell}}\right).$$

The new potential of GO in affecting cancerous cells is explored, as it has good surface functionality due to the oxidation process produced by using Hummer’s method. Hence, GO was chosen to observe the treatment effects on MCF7 breast cancer cells and MCF10a normal breast based on the cell viability and after the incubation period of 24 h and 48 h. Even though the studies elucidating the effects of GO on normal cells are very limited in comparing studies on GO impact on cancer cells, the precise mechanism that cause difference effect GO on normal and cancer cells is still unknown^13^. Therefore, in contrast to the previous research, the electrical responses and effects of MCF7 and MCF10a without using any redox solutions or biomarkers were studied to observe the direct interactions between the cells and GO. This is very important to fully understand the electrical responses between the cells, as it may be useful for developing an early breast cancer detection device.

## Experimental details

### Pre-oxidized graphite

The first step of the study was to prepare the pre-oxidized graphite by following the protocol reported by Ref.^14^. Firstly, a mixture was prepared by dissolving 3 g of graphite powder (NE Scientific, Malaysia), 2.5 g of potassium persulfate (P_2_S_2_O_8_) (Sigma-Aldrich, USA) and 2.5 g of phosphorus pentoxide (P_2_O_5_) (Sigma-Aldrich, USA) in 12 mL of sulfuric acid (H_2_SO_4_) (Sigma-Aldrich, USA). Then, the solution mixture was kept in an oil bath for four and a half hours under the temperature of 80 °C. Next, the mixture was cooled to the ambient temperature before it was diluted further with 500 mL of deionized (DI) water and left overnight. Finally, the mixture was filtered by using a 0.22 μm PTFE membrane and was washed with DI water to achieve neutral conditions through removing the residual acid.

### Modified Hummer’s method

This section described Hummer’s method to produce the aqueous GO with some modification. First, the pre-oxidized graphite was added into 120 mL of concentrated sulfuric acid pre-chilled to 0 °C and was kept incubated inside an ice bath. Then, 15 g of potassium permanganate (KMnO_4_) (Sigma-Aldrich, USA) was added gradually into the mixture to prevent extravagant heat. The mixture was stirred at 800 rpm and must be kept under 20 °C. Then, the mixture was stirred for 2 h under 35 °C. After that, the mixture was added with 250 mL of DI water, before being placed into the ice bath for another 2 h. Next, 700 mL of DI water was added, followed by 20 mL of 30% concentrated hydrogen peroxide (H_2_O_2_) (Sigma-Aldrich, USA). This reaction generated an aggressive spark, while the temperature rose to 70 °C. Meanwhile, the mixture changed to a yellowish color and indicated the production of well-oxidized graphite oxide. To remove the metal ions, the mixture was filtered and washed with 1:10 HCl solution, followed by 1 L of DI water to discard the residual acid. The resulting graphite oxide powder was dried at room temperature and weighed. Appropriate amount of GO powder was then dissolved with DI water to fix the concentration to 30 mg/mL.

### The production of different pH of GO

The aqueous GO solution was prepared into pH 5, by firstly using the titration method with the ratio of HCl: GO solution = 1:10. Next, the analyte solution was centrifuged at 6000 rpm for 30 min to obtain the concentrated graphite oxide. The separated supernatant liquid was removed and fresh DI water was added into the GO (the solid product) before the pH of the solution was measured (HI 98130 pH tester, HANNA Instrument, USA). The process of centrifugation, removing the supernatant liquid and adding fresh DI water was repeated for 16 times until pH 5 was achieved. For pH 6 and 7, those processes were repeated for 20 times and 24 times, respectively.

### The production of different concentration of GO

For this experiment, 5 mg/mL of GO with a pH of 5 was used to prepare a stock solution. Then, this GO stock solution was further diluted with DI water to produce 6 different concentrations; 2.5, 6.25, 12.5, 25, 50 and 100 μg/mL, respectively.

### GO characterization

The GO was characterized by using Raman spectroscopy (HORIBA Scientific, Xplora Plus, USA), X-ray Diffraction spectroscopy (XRD, Malvern Panalytical, Netherlands), Field Emission Scanning Electron Microscope (FESEM, Nova Nanosem 230, FEI, North America), Energy-Dispersive X-ray spectroscopy (EDX, Nova Nanosem 230, FEI, North America) and Thermogravimetric analysis (TGA, TGA-DSC HT 3, Mettler Toledo, USA) in order to validate and confirm the morphology and graphitic nature of GO.

### Cell culture

The breast cancer cell (MCF7) obtained from Advanced Medical and Dental Institute (AMDI, Universiti Sains Malaysia, USM) were cultured in RPMI 1640 medium (Gibco, USA) supplemented with 5% fetal bovine serum (FBS) (Gibco, USA) and 1% penicillin–streptomycin (Gibco, USA) were incubated in a 90% humidified atmosphere with 5% CO_2_ under 37 °C. The normal breast cell (MCF10a), also obtained from AMDI, (USM) were cultured in Dulbecco’s Modified Eagle Medium (DMEM) (Thermo Fisher Scientific, USA) supplemented with 10 μg/mL insulin (Thermo Fisher Scientific, USA), 20 ng/mL human epidermal growth factor (HEGF) (Thermo Fisher Scientific, USA), 0.5 µg/mL hydrocortisone (Thermo Fisher Scientific, USA), 5% horse serum (Thermo Fisher Scientific, USA) and 5% penicillin–streptomycin (Gibco, USA). The cells were also incubated in a 90% humidified atmosphere with 5% CO_2_ under 37 °C.

### Cell viability

MCF7 (1 × 10^4^ cells/mL) and MCF10a (1 × 10^4^ cells/mL) were seeded into 96-well plates for 24 h. Then, each well was added with either different pH (pH of 5, 6 and 7) and concentrations (concentration of 2.5, 6.25, 12.5, 25, 50 and 100 μg/mL) of GO, or with only medium. After that, the plates were incubated for an additional 24 and 48 h. After incubation, the medium was discarded, and 10 μL of PrestoBlue (Thermo Fisher Scientific, USA) reagent (final volume 100 μL, freshly diluted in RPMI and DMEM immediately before treatment) was added and incubated for another 1 h. The experiment for every condition of untreated and GO treated cells was conducted in triplicate for each 96-well plates and was repeated three times. The results were measured with the excitation wavelength set at 590 nm for the fluorescent signal detection mode, by using the FLUOstar Omega microplate reader (BMG LABTECH, Australia). The percentage number of viable cells was calculated by using the Eqs. (5) to (8) as shown below.

5 $${\text{Average }}\,{\text{media }} = \, \left( {{\text{summation}}\,{\text{of}}\,{\text{three}}\,{\text{well}}\,{\text{plates}}\,{\text{for}}\,{\text{media}}\,{\text{only}}} \right) \, \div { 3,}$$

6 $${\text{No}}.\,{\text{of}}\,{\text{cells }} = \, \left( {{\text{Untreated}}\,{\text{cell}}\,{\text{or}}\,{\text{treated}}\,{\text{cell}}\,{\text{for}}\,{\text{each}}\,{\text{well}}} \right) \, {-}{\text{ average}}\,{\text{media,}}$$

7 $${\text{Average}}\,{\text{untreated}}\,{\text{cells}} = \, \left( {{\text{summation}}\,{\text{of}}\,{\text{three}}\,{\text{well}}\,{\text{plates}}\,{\text{for}}\,{\text{untreated}}\,{\text{cells}}} \right) \, \div { 3,}$$

8 $${\text{No}}.\,{\text{of}}\,{\text{viable}}\,{\text{cells }}\left( \% \right) \, = \, \left( {{\text{Average}}\,{\text{treated}}\,{\text{cell }} \div {\text{ average}}\,{\text{untreated}}\,{\text{cell}}} \right) \, \times { 1}00 \, \% .$$

### Electrical characterization

The electrical responses of MCF7 and MCF10a cells on IDE were investigated by using an LCR meter (Agilent E4980 20 Hz–2 MHz Precision LCR (Inductance, Capacitance, Resistance) meter, USA). Briefly, the cell’s medium was firstly removed, then MCF7 and MCF10a with and without the GO treatment were trypsinized and the reaction was terminated by adding newly fresh medium. The cells in suspension were then centrifuged, in order to get the cell’s pellets. Then, 1.5 mL of medium was added and the cells were manually counted using hemocytometer. Lastly, 5 µL of the MCF7 and MCF10a cells were deposited onto the IDE by using the drop cast method. The Z_cell_ and resistance changes before and after deposition were then monitored at a frequency between 20 Hz to 2 MHz using the LCR meter.

## Results and discussion

### GO characterization

**Figure 1:**
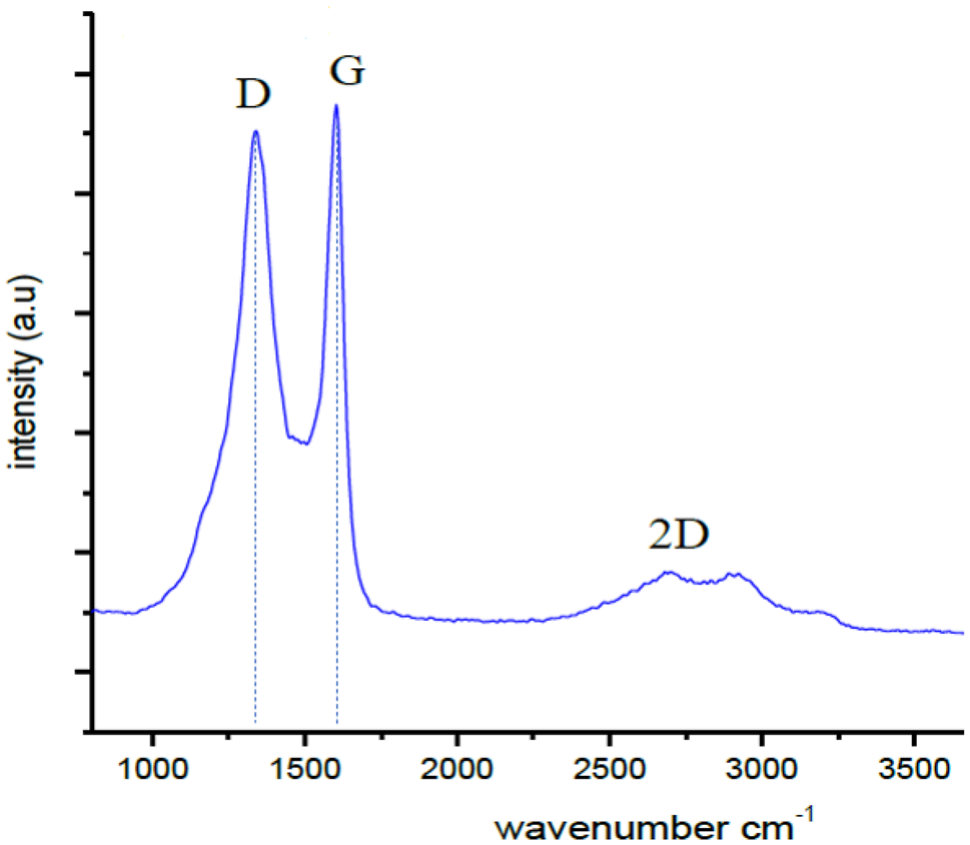
Raman spectrum of GO shows the present of D peak at 1377 cm^−1^ and G peak at 1580 cm^−1^ and 2D peak at 2700 cm^−1^.

The obtained GO was first characterized by using the Raman spectroscopy, XRD spectroscopy, FESEM, EDX spectroscopy and Thermogravimetric analysis (TGA) to confirm the graphitic nature, crystal structure, morphology, composition of elements and decarboxylation process respectively. Firstly, Raman spectroscopy was used in order to study the defects and the structure’s order of the GO. According to graphitic nature, the D and G peaks can be detected in the range of 1200–1500 cm^−1^ and 1500–1800 cm^−1^ wavenumber respectively. From the image obtained by using the Raman spectroscopy, the presence of a defect characteristic, D peak at 1377 cm^−1^ (as shown in Fig. 1) can be seen. The peak may be produced by the disordered bands in sp^2^ hybridized carbon materials and led to the disruption of the symmetrical hexagonal graphitic lattice as a result of edge defects, internal structural defects and dangling bonds^15^. Meanwhile, the G peak was centered at 1580 cm^−1^ and was referred to as the first order of the Raman band creating a D to G ratio of 0.87. This may be caused by all the sp^2^ hybridized carbon materials that were related to the C–C vibrational mode^16^. The shift in the G band from 1582 cm^−1^ to 1599 cm^−1^ of GO was due to the presence of isolated double bonds on the GO carbon network^15^. Finally, the 2D peak was also detected at 2700 cm^−1^.

**Figure 2:**
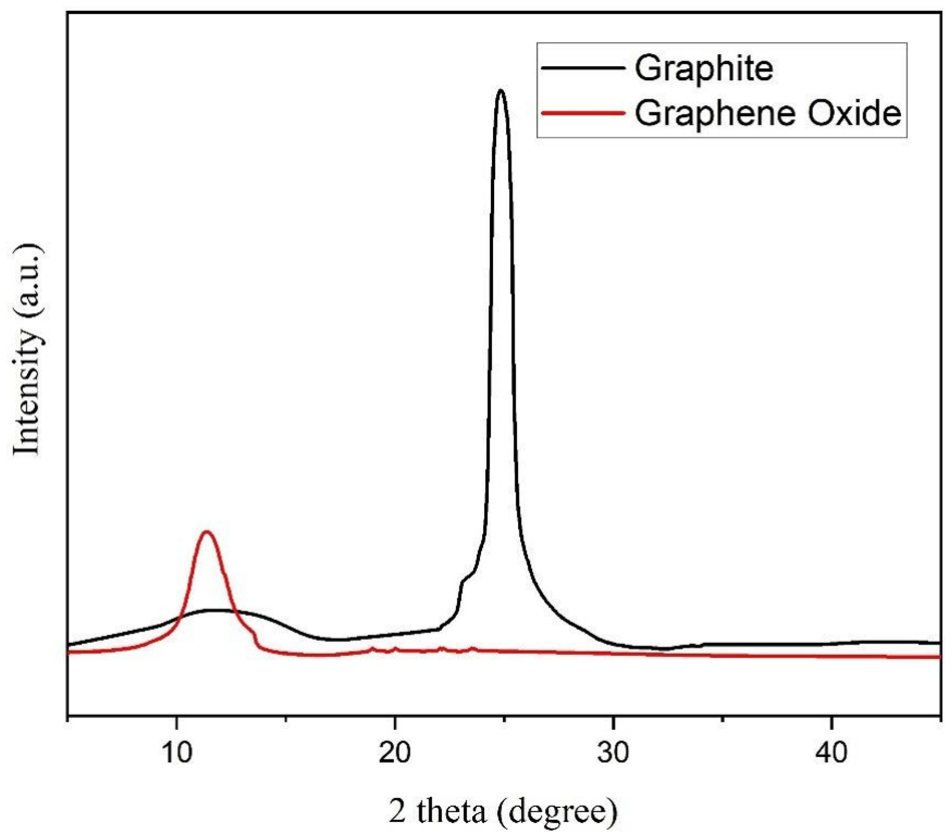
XRD patterns of graphite and GO deposited on Si with the peak of graphite observed at 2Ɵ = 26.73° and the peak of GO at 2Ɵ = 11.54°.

Next, the X-Ray Diffraction (XRD) was used to identify the phase identification of the crystalline structure in GO. Before conducting the test, the GO solution was first deposited onto the Silicon Dioxide (SiO_2_) substrate by using a drop cast method. The XRD pattern in Fig. 2 was comparable to that obtained by Ref.^16^, confirming the crystalline nature of graphite and GO. In the data, graphite exhibited a sharp peak at 2Ɵ = 26.73 degrees with d-spacing at 3.33 Å. After the oxidation of graphite, the sharp reflection peak was shifted to the lower angle at 2Ɵ = 11.54 degrees with d-spacing at 7.67 Å, due to the formation of oxygen functional groups and the intercalation of water molecules into the carbon layer structure^17^.

**Figure 3:**
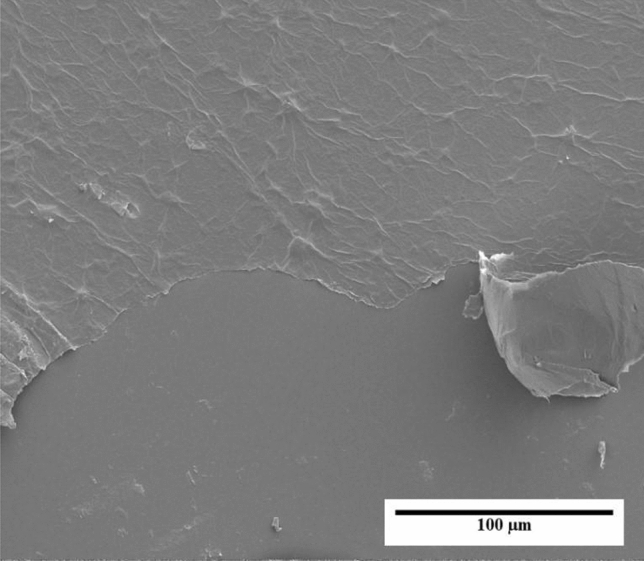
FESEM of thin GO monolayer made up of folded and wrinkled graphene films.

For Field Emission Scanning Electron Microscopy observation, the FESEM was operated at 15 kV and at a constant working distance of 4.9 mm to produce the optimal imaging conditions. This analysis is primarily used to determine the surface morphology at high magnification. From the observation analysis of Fig. 3 showed that the GO is a very thin monolayer or few-layered structures made up of folded and wrinkled graphene films. The films were thin due to the mechanical forces produced by using an ultrasonication bath. The films were wrinkled, folded and stacked by a few layers of graphene due to the strong π–π interaction at the surfaces^18^.

**Figure 4:**
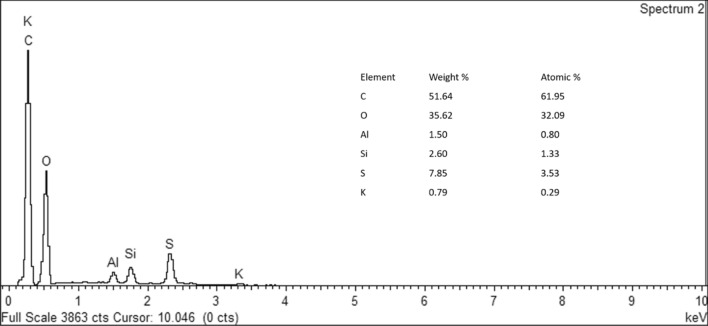
EDX spectra of GO deposited on Si shows the two most high atomic percentage which is Carbon (C) and Oxygen (O) at 51.64% and 35.62% respectively.

The Energy Dispersive X-Ray (EDX) measurement was used to investigate the elemental and quantitative compositions of the materials. In this measurement, an accelerating voltage of 20 kV was used with a scan time of 100 s per sampling area. The EDX spectrum of GO is as shown in Fig. 4. The observation revealed the presence of Carbon (C), Oxygen (O), Aluminum (Al), Silicon (Si), Sulphur (S) and Potassium (K). The C content of the GO was valued at 51.64% and was obviously the highest as graphite is a carbon material. This was followed by 35.62% of O content due to the oxygen-containing functional groups produced from the oxidation process by using Hummer’s method. The mass ratio of O/C was 0.68. Lastly, the content of S, Si, Al and K were reported at 7.85%, 2.60%, 1.50% and 0.79% respectively.

**Figure 5:**
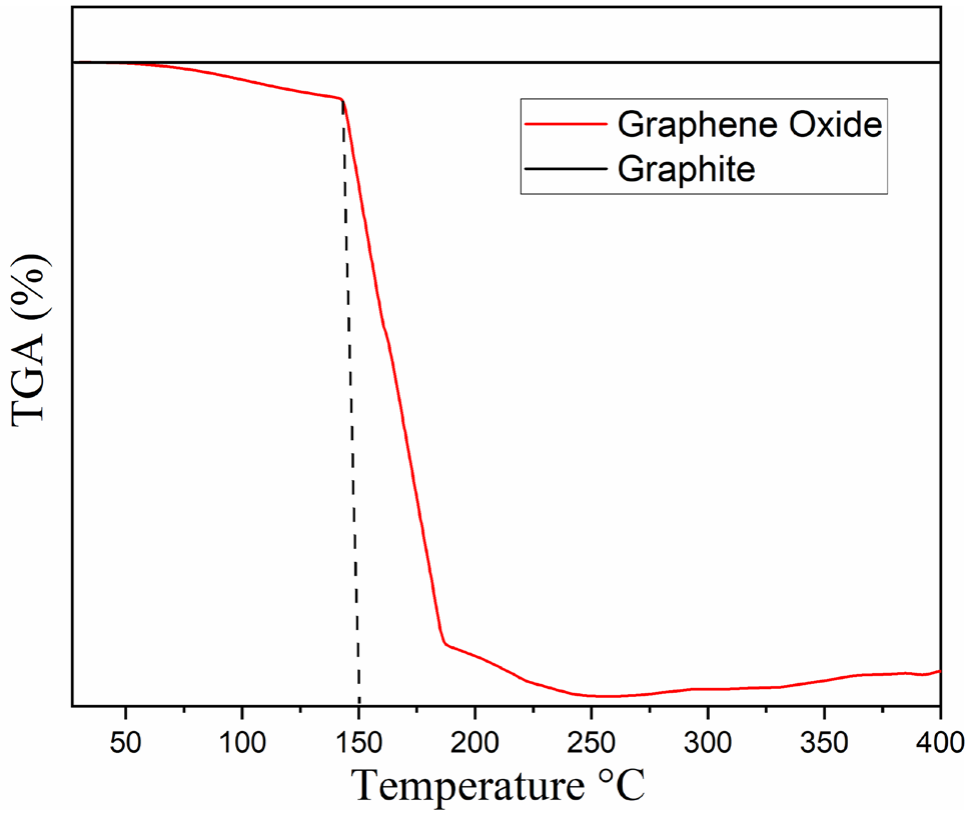
TGA analysis curves of graphite and graphene oxide (GO).

Thermogravimetric analysis (TGA) was used to determine the temperature at when a material was completely decomposed. The drop in the mass shows the material was undergoing the decomposition process. Based on Fig. 5 below, a TGA comparison between graphite and GO can be seen. While graphite percentage mass maintained close to 100% TGA because of a non-volatile nature, GO showed drop throughout the process mainly once the temperature hit 150 °C. This finding can be related with a study done by Ref.^19^. According to them, the majority of the carbon atoms in GO have been converted from graphitic sp^2^ to a non-graphitic sp^3^ hybridized carbon that contains high density of defects due to the oxidation process that occurred. The defects and weaker interaction between the exfoliated graphene layers cause the thermal degradation temperature to reach much faster. Hence the difference between the stability in mass percentage between graphite and GO can clearly be observed.

### Cell viability and pH of GO

**Figure 6:**
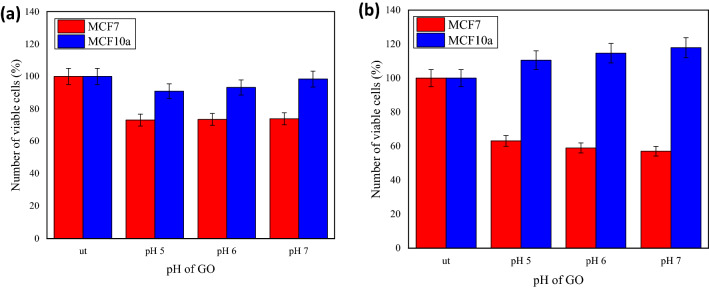
The comparison between the number of viable cells of MCF7 and MCF10a against pH of GO after (**a**) 24 h (**b**) 48 h. The ut represents the untreated cells or cells which were not treated with GO.

One of the components that can be taken into consideration when maintaining the solubility of GO is the pH of the prepared GO. According to Refs.^20,21^, the carboxyl groups of GO at lower pH are protonated, thus making the GO less hydrophilic, while at the higher pH of GO, the carboxyl groups are deprotonated, eventually enhance the hydrophilic features and resulting in better GO solubility. The cell membranes are semipermeable and allow selected molecules to pass through the barriers and induce changes to the biological interactions. Therefore, to distinguish the interaction between the MCF7 breast cancer cells and MCF10a normal breast cells due to the presence of GO, an observation on the effect of different pH of GO towards the cell viability was done. Figure 6 shows the numbers of viable MCF7 and MCF10a cells against the pH of GO after 24 h (as shown in Fig. 6a) and 48 h (as shown in Fig. 6b) incubation. After 24 h of incubation (i.e. incubation period) for MCF10a (Fig. 6a), the percentage number of viable cells decreased to 90.5%, 92.8% and 98.4% after the GO treatments with the pH 5, 6 and 7, respectively. From the percentage obtained, only a slight reduction in the cell’s numbers can be seen. The pH value for normal cells usually ranging from pH 7.2 to pH 7.5 which is in a neutral condition^22^. Hence, when the MCF10a surrounding was slightly acidic due to the pH value of GO at pH 5 and pH 6, more reduction in the cell viability can be seen. Meanwhile, after 48 h of incubation, the cell viability increased significantly to 109.8%, 113.6% and 116.4%, respectively at the pH 5, 6 and 7, as compared to after 24 h incubation. A paper by Ref.^23^ discussed that normal human cells undergo proliferating on average every 24 h. During this division timing, the cells were allowed to synchronize with the physiological process and the changes in their environment and hence suggested the best maximum effect to be measured at 24 h incubation period to prevent any interruption of other external factors.

As shown in Fig. 6a, the percentages of viable MCF7 cells treated with different GO pH for 24 h was relatively similar and were approximately 73.13%, 73.53% and 73.93%, respectively. This result shows greater reduce in the cell viability compared to the normal breast cells MCF10a suggesting that GO gave greater effects towards the breast cancer cells MCF7 than the normal breast cells MCF10a. Plus, when the incubation period was increased to 48 h (as shown in Fig. 6b), the percentages of viable MCF7 cells treated with different GO pH of 5, 6 and 7 dropped further to 63.07%, 58.93% and 56.98%, respectively. These continuous decreases with respect to the incubation time somehow only occurred for the MCF7 compared to the MCF10a and was supported by Ref.^24^ that stated GO have the ability to hinders the proliferation of the cancer stem cells in wide array of cancer and is not toxic to the normal pluripotent stem cells, but stimulate their differentiation into various cell types. Moreover, the GO of pH 7 had a greater effect on the cell viability, than the GO of pH 5, and this corresponded to the hydrophilic properties. As the pH increased, the hydrophilicity of GO also increased^21^. Hence, the prepared GO resulted in better solubility for better interactions with the cells. Therefore, for the next dose-dependent cell viability study, the GO of pH 7 was selected to be tested with varying GO concentrations, as the GO of pH 7 was shown to inhibit the growth of breast cancer MCF7 cells while maintaining or increasing the viability of normal breast MCF10a cells.

**Figure 7:**
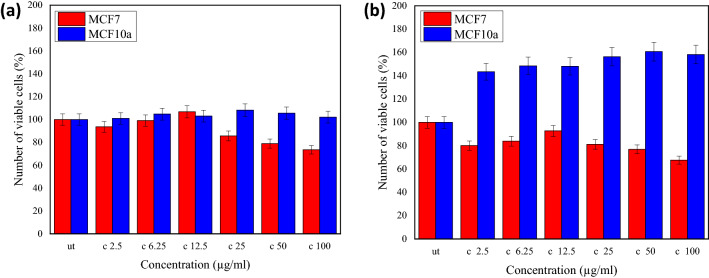
The comparison between the number of viable cells of MCF7 and MCF10a against concentration of GO after (**a**) 24 h and (**b**) 48 h. The ut represents the untreated cells or cells which were not treated with GO.

For the dose-dependent cell viability study, the GO concentrations were varied into six different concentrations (2.5, 6.25, 12.5, 25, 50 and 100 µg/mL) while the pH of GO was fixed at 7. Figure 7 shows the comparison between the number of viable MCF10a and MCF7 cells against the different concentrations after 24 h incubation time. The graph in Fig. 7a shows a small percentage difference between the number of untreated and treated MCF10a cells. The average number of MCF10a cells varied between 1 to 7.89%, as compared to the untreated MCF10a cells. Here, the value larger than 100% was inferred to the activation of MCF10a. In the case of a 48 h incubation period, the results, as in Fig. 7b, showed that the percentages of viable MCF10a were much larger (ca. 43.47–60.6%), as compared to the untreated MCF10a cells. At the higher concentrations of GO (> 25 µg/mL), the percentages of viable MCF10a cells were slightly larger than that of the lower GO concentrations. This increase resembled a much higher activation of MCF10a cells. Overall, the MCF10a activation does not have a strong dependency on the GO concentrations but rather is dependent on the incubation time.

In the case of GO incubation with the MCF7 cells, the data in Fig. 7a showed the number of viable cells was slightly proportional to the GO concentrations of less than 25 µg/mL, then showed inversely proportional relation appeared for the concentrations of more than 25 µg/mL. Moreover, at 12.5 µg/mL, the number of viable cells was larger than 100%, thus indicating the activation of cells. The results were insignificant by considering the 10% error bar. However, the GO concentrations of less than 25 µg/mL are not sufficient to increase the inhibition rate, similar to the study for GO reacting with the cancer cells^25^.

After the 48 h incubation period, a similar trend of data was observed. However, the difference between the number of viable MCF7 and MCF10a cells was much larger at the higher GO concentration. For example, at the GO concentration of 100 µg/mL, the difference was ca. 30% for the 24 h incubation period, while it was 90% for that 48 h, hence was proportional to the incubation time. GO increased MCF7 toxicity, while it has much lower toxicity to normal breast epithelial cells, MCF10a. One of the possible mechanisms that cause cytotoxicity effect is the eliciting of apoptosis such that the GO stimulated molecular and cellular apoptosis in cancer cells whereas demonstrated low apoptosis observed in treated normal breast epithelial cells^26^. Nevertheless, the focus of current study is on the phenomenological characterization of MCF7 and MCF10a response to GO in terms of viability and electrical response, which paving the way to a more in-depth study such as the molecular dynamics studies that could explain the effect of GO on the two cell lines^27^.

### Electrical responses characterization

The MCF7 and MCF10a cells, untreated and treated with different GO pH for 24 and 48 h were characterized based on the electrical response. Gold interdigitated electrodes on glass substrate with 10 µm gaps having initial resistance of 2.0 × 10^5^ Ω were used in this measurement. A constant value of 1 V was supplied to the electrode from the LCR meter (Agilent E4980 20 Hz–2 MHz Precision LCR (Inductance, Capacitance, Resistance) meter, USA) equipped with LabVIEW 2012 software, to obtain the electrical parameters values. The measurement was done at frequency between 20 Hz to 2 MHz.

#### The electrical impedance and resistance responses of untreated and treated MCF7 and MCF10a cells with different pH of GO after 24 h

**Figure 8:**
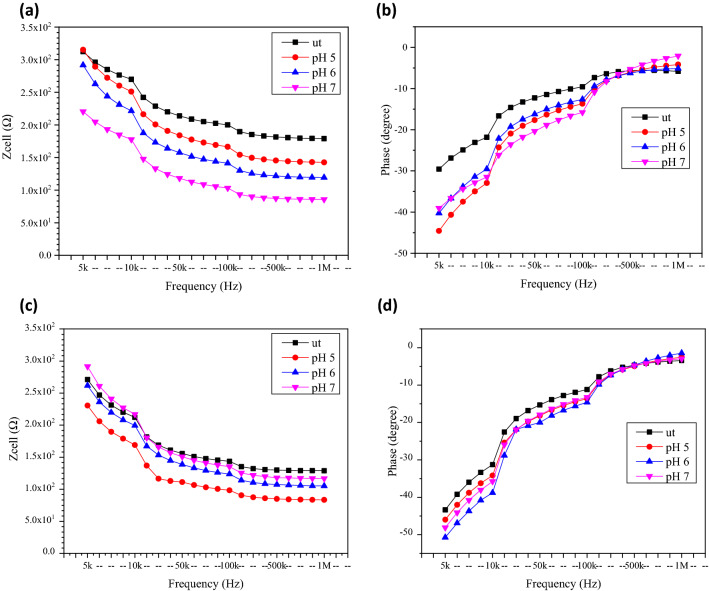
The electrical (**a**) Z_cell_ and (**b**) phase of untreated and treated breast cancer cell (MCF7) and the electrical (**c**) Z_cell_ and (**d**) phase of untreated and treated normal breast cell (MCF10a) with different pH of GO for 24 h.

Figure 8a,b shows the electrical Z_cell_ and phase values from the untreated and treated MCF7 cells with different GO pH for 24 h. From the plot, the MCF7 cells treated with GO of pH 5 showed the highest Z_cell_ value, while the cells treated with GO of pH 7 showed the lowest Z_cell_ value. For the MCF7 cells treated with GO of pH 5, the Z_cell_ value measured was 315.25 Ω with phase of − 44.58° at the frequency of 5 kHz and dropped to 220.45 Ω with phase of, − 39.05° after the GO treatment at the pH of 7. The opposite was observed in the MCF10a. From the results shown in Fig. 8c,d, the MCF10a cells treated with GO at the pH of 7 produce highest Z_cell_ and cells treated with GO at pH 5 show the lowest impedance. The Z_cell_ and phase measure at 5 kHz for MCF10a treated with GO at pH 7 was 291.52 Ω with a phase of − 48.10°, while the MCF10a cells treated with GO at the pH of 5 at the same frequency showed the lower Z_cell_ value of 230.58 Ω with a phase of − 45.98°. The overall value of measured Z_cell_ of the MCF7 was slightly higher than the measured Z_cell_ of the MCF10a with decreasing trend as frequency increased observed in both samples. The phase for all samples was also measured in the negative values. The negative phase was due to the characteristic of the cell membranes which is insulative causing it to have capacitive effect.

As stated by Ref.^12^, without the cells, the Z_cell_ of the system can only come from the very low electrode capacitance (C_dl_) and the solution resistance (R_sol_)_._ For the MCF7 medium (RPMI), the R_sol_ value was 2.49 kΩ with the C_dl_ equals to 989 nF, while for the MCF10a medium (DMEM), the R_sol_ value was 1.63 kΩ with the C_dl_ equals to 1420 nF. After dropping the treated and untreated cells onto the electrode surface, the Z_cell_ of the attached cells is modelled as the capacitance component (C_cell_) and the resistance component (R_cell_)^12,28^.

Table 1 shows the R_cell_ and C_cell_ of untreated and treated MCF7 and MCF10a with different pH of GO for 24 h. For the untreated cells, the MCF7 produces R_cell_ value (4732.9 Ω) higher than MCF10a (2467.2 Ω) with C_cell_ value of 775 nF and 710 nF respectively. The MCF7 shows decreased in R_cell_ and increased in C_cell_ value as the pH was increased from 5 to 7. The reverse was observed in the MCF10a where the value of R_cell_ increased and C_cell_ decreased with increasing pH value. The R_cell_ value can be attributed to a few factors such as cell viability, cell types and pH of the solution. For MCF7 the decreasing trend in the R_cell_ as the pH of GO increase was attributed mostly due to the pH of the solution as the difference in cell viability between pH was not differ by much. As for the MCF10a, the cell viability increased slightly with increasing pH of the GO. Cell membranes have insulating behavior. The increase in cell numbers will contribute to the increase of R_cell_ as the insulating comportment of the membranes reduces the current flow. This will also contribute to decline in the capacitance values due to the increased number of highly insulating cell membranes of viable cells that contributed to the increasing Z_cell_, as the capacitance was inversely related to the impedance^12,28–30^.

**Table 1:** The resistance and capacitance of MCF7 and MCF10a before and after were treated with different pH of GO for 24 h incubation time. ut: Untreated cells or cells which was not treated with GO.

pH of GO	MCF7		MCF10a	
	Resistance of component, R_cell_ (Ω)	Capacitance of component, C_cell_ (nF)	Resistance of component, R_cell_ (Ω)	Capacitance of component, C_cell_ (nF)
ut	4732.90	775.0	2467.20	710.0
pH 5	5370.97	235.0	4819.82	330.0
pH 6	4818.53	276.0	5010.24	329.0
pH 7	3857.77	814.0	6398.32	267.0

The results of Z_cell_ (as shown in Fig. 8) and resistance (as shown in Table 1) were compared with the number of viable cells (as shown in Fig. 6). The findings demonstrated that the MCF7 breast cancer cells had higher cell viability and lower resistance value as the pH increased, while the MCF10a also showed a similar increasing trend in cell viability and resistance value as the pH increased. It has been verified by Ref.^31^ that the smaller gaps between the cells and electrodes led to better sensitivity in the cells’ electrical impedance signals and resistances.

#### The electrical impedance and resistance responses of untreated and treated MCF7 and MCF10a cells with different pH of GO after 48 h

**Figure 9:**
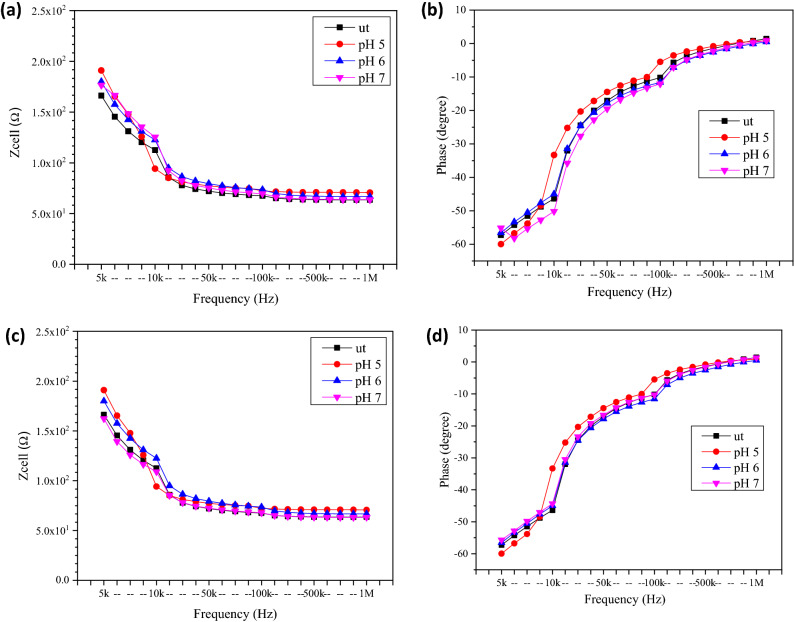
The electrical (**a**) Z_cell_ and (**b**) phase of untreated and treated breast cancer cell (MCF7) and the (**c**) Z_cell_ and (**d**) phase of untreated and treated normal breast cell (MCF10a) with different pH of GO for 48 h.

Figure 9 shows the electrical Z_cell_ and phase ranging over the frequency ranging 5 kHz to 1 MHz for untreated and treated MCF7 and MCF10a with different GO pH for incubation period of 48 h. The results for both cells shows that the Z_cell_ decreased as the frequency increased and it converged at the higher frequencies. According to Ref.^12^, this condition was due to the frequency dependent characteristics, as calculated according to Eq. (1). At 5 kHz, for MCF7 the Z_cell_ measure decreased from 191.01 Ω with phase of − 59.97° after the treatment with GO of pH 5 to 162.30 Ω with phase of − 55.14° after the treatment with GO of pH 7. Meanwhile, for MCF10a at the same frequency, the Z_cell_ increased from 131.39 Ω with the phase of − 47.46° after the treatment with GO of pH 5 to 170.29 Ω with the phase of − 57.70° after the treatment with GO of pH 7. The value of measured Z_cell_ of the MCF7 was generally higher than the measured Z_cell_ of the MCF10a. It was also evident that only small Z_cell_ variation was observed between the ut and sample with different pH value. The phase measure for all samples was in the negative region corresponding to the capacitive behavior of the cell membranes and could be considered as inherent characteristics of cell membranes which act as dielectric layer.

The R_cell_ and C_cell_ for both the treated MCF7 and MCF10a cells were also compared, and the results are as tabulated in Table 2. The R_cell_ value for MCF7 cells was the lowest at 3.23 kΩ, with the C_cell_ equals to 673 nF, while the R_cell_ value MCF10a was the highest at 3.59 kΩ, with the C_cell_ equals to 847 nF, after the treatment with GO of pH 7. The result for the Z_cell_ (as shown in Fig. 9) and R_cell_ (as shown in Table 2) were compared with the number of viable cells (as shown in Fig. 6).

**Table 2:** The resistance and capacitance of MCF7 and MCF10a before and after treated with different pH of GO for 48 h incubation time. ut: Untreated cells or cells which was not treated with GO.

pH of GO	MCF7		MCF10a	
	Resistance of component, R_cell_ (Ω)	Capacitance of component, C_cell_ (nF)	Resistance of component, R_cell_ (Ω)	Capacitance of component, C_cell_ (nF)
ut	2944.70	1140.0	2427.30	1130.0
pH 5	3319.37	463.0	2350.95	1240.0
pH 6	3257.15	642.0	2454.77	1170.0
pH 7	3226.02	673.0	3589.18	847.0

From the results it was observed that the MCF10a has higher C_cell_ as compared to MCF7 at respective pH of GO. This could be due to the lower capacitive behavior of the cell membrane for metastatic grade cancer, which could be caused by their low sterol and phospholipid contents^32^. The irregular shape of MCF7 as compared to MCF10a can cause it to become less inflexible, which contributes to lower polarization which also led to lower capacitance value^33^.

It was also observed that the MCF7 has higher R_cell_ value decreased as pH value increased while for MCF10a the R_cell_ increases as pH value increased. This can be related to the viability of the cells after 48 h. In MCF7 cell viability reduced to 56.98% from 63.07% as pH of GO increased from 5 to 7. In comparison to MCF10a in which the cell viability improves from 109.8 to 116.4% cells viability. Cell membrane is made up of a highly mobile lipid molecule bilayer that is an electrically insulator^34^. Having more cells available will further increase resistance. The more cells available will further decrease the gap between the highly insulating cell membranes of viable cells with the electrode surface and the decreased capacitance^35^.

#### The electrical impedance and resistance responses of untreated and treated MCF7 and MCF10a cells with different concentrations of GO after 24 h

**Figure 10:**
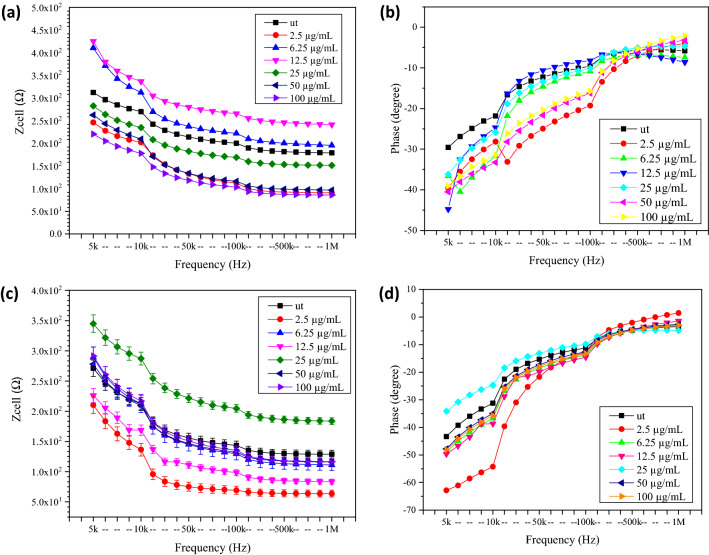
The electrical (**a**) Z_cell_ and (**b**) phase of untreated and treated breast cancer cell (MCF7) and the (**c**) Z_cell_ and (**d**) phase of untreated and treated normal breast cell (MCF10a) with different concentration of GO for 24 h.

The dose dependent GO effects on the MCF7 and MCF10a cells are shown in Fig. 10a,b. The findings showed that the Z_cell_ of MCF7 cells rose after treatment with 12.5 µg/mL of GO at the pH of 7. However, the Z_cell_ of MCF7 cells decreased as the GO concentrations increased. At a frequency of 5 kHz, the electrical Z_cell_ of the MCF7 cells treated with 12.5 µg/mL of GO was 425.27 Ω with a phase of − 44.78°, while for the MCF7 cells treated with 100 µg/mL of GO, the electrical Z_cell_ was 220.45 Ω with a phase of − 39.05°. From Fig. 10c,d, the MCF10a cells treated with 25 µg/mL of GO showed the highest Z_cell_ value of 345.03 Ω with a phase of − 34.13°, while the MCF10a cells treated with 2.5 µg/mL of GO showed the lowest Z_cell_ of 210.27 Ω with a phase of − 62.83° at 5 kHz.

An increasing Z_cell_ value shows the increasing resistance of the cells. For the MCF7 treated with 12.5 µg/mL of GO, the highest R_cell_ value was 7.73 kΩ with the C_cell_ equals to 256 nF. Meanwhile, for the MCF10a treated with 25 µg/mL of GO, the highest resistance value was 6.62 kΩ with the C_cell_ equals to 225 nF. The resistance and capacitance results of MCF7 and MCF10a untreated and treated with different GO concentrations for 24 h were summarized in Table 3.

**Table 3:** The resistance and capacitance of MCF7 and MCF10a before and after treated with different concentrations of GO for 24 h incubation time. ut: Untreated cells or cells which was not treated with GO.

Concentration of GO (µg/mL)	MCF7		MCF10a	
	Resistance of component, R_cell_ (Ω)	Capacitance of component, C_cell_ (nF)	Resistance of component, R_cell_ (Ω)	Capacitance of component, C_cell_ (nF)
ut	4732.90	775.0	2467.20	710.0
2.5	4497.88	716.0	4819.82	935.0
6.25	4818.53	276.0	6219.79	288.0
12.5	7730.03	256.0	5078.66	687.0
25	4451.92	589.0	6621.56	225.0
50	3257.91	640.0	5835.85	261.0
100	3857.77	814.0	6398.32	267.0

The Z_cell_ and resistance results trends were correlated to the number of viable cells trend, as shown in Fig. 7a. The highest number of viable MCF7 cells was recorded after the treatment with 12.5 µg/mL of GO, while the lowest was recorded after the treatment with 100 µg/mL of GO. Meanwhile, the highest number of viable MCF10a was recorded after the treatment with 25 µg/mL of GO, while the lowest was recorded after the treatment with 2.5 µg/mL of GO. The trend was comparable to the findings reported by Ref.^35^. The study observed that the Z_cell_ and resistance increased to correspond with the decreased gap between the highly insulating cell membranes of viable cells with the electrode surface; hence it was related to the decreased capacitance. When the capacitance decreased, the Z_cell_ value increased, similar to the resistance value.

#### The electrical impedance and resistance responses of untreated and treated MCF7 and MCF10a cells with different concentrations of GO after 48 h

**Figure 11:**
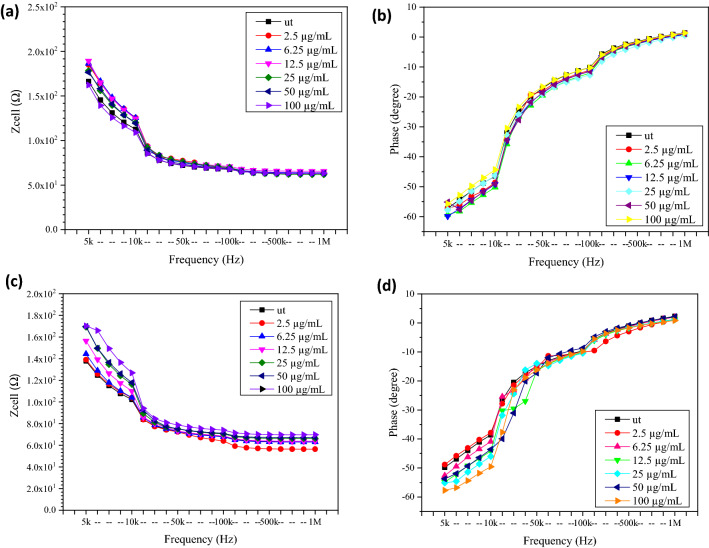
The electrical (**a**) Z_cell_ and (**b**) phase of untreated and treated breast cancer cell (MCF7) and the (**c**) Z_cell_ and (**d**) phase of untreated and treated normal breast cell (MCF10a) with different concentration of GO for 48 h.

Figure 11 shows the electrical Z_cell_ and phase of MCF7 and MCF10a untreated or treated with different GO concentrations, with a longer incubation period of 48 h. At a frequency below 500 kHz, the Z_cell_ decreased as the frequency increased. In contrast, it intersected and became stable at frequencies higher than 500 kHz. According to Ref.^12^, this condition was due to the frequency-dependent characteristics, as calculated according to Eq. (1). For the MCF7 treated with 100 µg/mL of GO, the lowest Z_cell_ was 162.30 Ω, with the phase of − 55.68°, while the highest Z_cell_ was 189.24 Ω, with the phase of − 59.87° at the frequency of 5 kHz. On the other hand, for the MCF10a, the Z_cell_ increased from 139.13 Ω with the phase of − 48.82° to 170.29 Ω with the phase of − 57.70° at 5 kHz after treated with a higher GO concentration of 100 µg/mL.

Table 4 shows the resistance (R_cell_) and capacitance (C_cell_) of MCF7 and MCF10a before and after incubation treatment with different GO concentrations for 48 h. For the MCF7, the lowest R_cell_ value was 3.23 kΩ, with the C_cell_ equals to 673 nF after treatment with 100 µg/mL of GO, while the highest R_cell_ value was 3.68 kΩ, with the C_cell_ equals to 563 nF after treatment with 12.5 µg/mL of GO.

**Table 4:** The resistance and capacitance of MCF7 and MCF10a before and after treated with different concentrations of GO for 48 h incubation time. ut: Untreated cells or cells which were not treated with GO.

Concentration of GO (µg/mL)	MCF7		MCF10a	
	Resistance of component, R_cell_ (Ω)	Capacitance of component, C_cell_ (nF)	Resistance of component, R_cell_ (Ω)	Capacitance of component, C_cell_ (nF)
ut	2944.70	1140.0	2427.30	1130.0
2.5	3648.55	631.0	2433.77	1141.0
6.25	3673.58	572.0	2509.06	1120.0
12.5	3684.56	563.0	2969.99	951.0
25	3486.65	601.0	2996.11	937.0
50	3276.41	628.0	3415.25	884.0
100	3226.02	673.0	3589.18	847.0

The Z_cell_ and resistance trends of MCF7 were similar for 24 and 48 h, as compared to the results presented in Fig. 9 and Table 3. Thus, the results proved that the Z_cell_ and resistance were increased linearly with an increasing amount of highly insulating viable cell membrane on the IDEs, subsequently leading to a decrease in the capacitance. These findings were in agreement with the findings of Ref.^12^. Meanwhile, the R_cell_ increased steadily from 2.43 to 3.59 kΩ, with the C_cell_ equals to 1143 nF and 847 nF for MCF10a after 100 µg/mL of GO treatment. It showed that the resistance trend was corresponding to the impedance, as shown in Fig. 11, and was comparable with the trend for the number of viable MCF7 and MCF10a cells, as shown in Fig. 7b.

## Conclusion

For this research, a few layers of GO, with an average size of 0.56–0.96 µm and an average thickness of 1.24–1.32 nm, were successfully produced by using the Hummer’s method. Then, the GO was further synthesized and characterized by using the Raman spectroscopy, XRD spectroscopy, FESEM, EDX spectroscopy and TGA analysis. The cell viability for both the MCF7 and MCF10a before and after treatment with GO was successfully determined by using the PrestoBlue cell viability assay. After treating both the MCF7 and MCF10a cells with three different pH (e.g., pH 5, 6 and 7), it was that when the GO pH increased to 7, the number of MCF7 viable cells was decreased, while the number of MCF10a viable cells was maintained at 24 h incubation time. Also, the number of MCF7 viable cells was further decreased with the increasing number of MCF10a viable cells at 48 h incubation time. Hence, it can be deduced that the GO of pH 7 was the most suitable pH to inhibit the MCF7 proliferation compared to pH 5 and pH 6, with the optimum incubation period at 24 h. In terms of the dose-dependent interaction between cells and GO, the concentration of GO was varied into six different concentrations (e.g., 2.5, 6.25, 12.5, 25, 50 and 100 µg/mL). For the electrical characterization part, the electrical properties of MCF7 and MCF10a cells before and after exposures to GO were successfully demonstrated by using 10 µm-gaps gold interdigitated electrodes connected to the LCR meter. In conclusion, the trend showing the number of viable cells was comparable with the capacitance, impedance, and resistance results. Thus, it was proven that the capacitance reduction was due to the increase in highly insulating MCF7 and MCF10a cells membrane. When dropped on the electrode surface, the highly insulated cells membrane caused an increase in electrical Z_cell_ and resistance. In conclusion, the electrical characterization and cell viability analyses of breast cancer cells using the GO deposited on IDE were successful.
